# iPSC-Derived MSCs Versus Originating Jaw Periosteal Cells: Comparison of Resulting Phenotype and Stem Cell Potential

**DOI:** 10.3390/ijms21020587

**Published:** 2020-01-16

**Authors:** Felix Umrath, Marbod Weber, Siegmar Reinert, Hans-Peter Wendel, Meltem Avci-Adali, Dorothea Alexander

**Affiliations:** 1 Department of Oral and Maxillofacial Surgery, University Hospital Tübingen, 72076 Tübingen, Germany; felix.umrath@med.uni-tuebingen.de (F.U.); Siegmar.reinert@med.uni-tuebingen.de (S.R.); 2 Department of Thoracic and Cardiovascular Surgery, University Hospital Tübingen, 72076 Tübingen, Germany; marbod.weber@uni-tuebingen.de (M.W.); hans-peter.wendel@med.uni-tuebingen.de (H.-P.W.); meltem.avci-adali@uni-tuebingen.de (M.A.-A.)

**Keywords:** iPSC-derived mesenchymal stem/stromal-like cells (iMSCs), induced pluripotent stem cells (iPSCs), jaw periosteal cells (JPCs), self-replicating RNA, differentiation, bone-tissue engineering

## Abstract

Induced pluripotent stem cell-derived mesenchymal stem cell-like cells (iMSCs) are considered to be a promising source of progenitor cells for approaches in the field of bone regeneration. In a previous study, we described the generation of footprint-free induced pluripotent stem cells (iPSCs) from human jaw periosteal cells (JPCs) by transfection of a self-replicating RNA (srRNA) and subsequent differentiation into functional osteogenic progenitor cells. In order to facilitate the prospective transfer into clinical practice, xeno-free reprogramming and differentiation methods were established. In this study, we compared the properties and stem cell potential of the iMSCs produced from JPC-derived iPSCs with the parental primary JPCs they were generated from. Our results demonstrated, on the one hand, a comparable differentiation potential of iMSCs and JPCs. Additionally, iMSCs showed significantly longer telomere lengths compared to JPCs indicating rejuvenation of the cells during reprogramming. On the other hand, proliferation, mitochondrial activity, and senescence-associated beta-galactosidase (SA-β-gal) activity indicated early senescence of iMSCs. These data demonstrate the requirement of further optimization strategies to improve mesenchymal development of JPC-derived iPSCs in order to take advantage of the best features of reprogrammed and rejuvenated cells.

## 1. Introduction

A growing population and aging societies lead to a rising demand for regenerative therapies in the field of orthopedic and maxillofacial surgery. In addition to classical treatments, cell therapies and tissue engineering approaches are pushing their way into clinical applications. For these purposes, mesenchymal stem/stromal cells (MSCs) isolated from different tissues are being tested in clinical trials [1]. Based on their stem cell potential, homing activity, and immunomodulatory properties, MSCs can be applied for the treatment of a variety of diseases [2,3,4]. Unfortunately, restricted availability, limited in vitro proliferation capacity, and limited differentiation potential impede their application in clinical routine [5]. In addition, it is particularly unfavorable that the quantity of MSCs in donor tissues decreases with age [6] as older patients make up the majority of the patients in need of such treatments.

For bone regeneration in the oral and maxillofacial region, it is obvious to choose the best suitable stem cell source, especially considering the strong mechanical load in this area. Convinced that jaw periosteal cells (JPCs) represent an optimal stem cell source, the goal of our work is to characterize this cell type in detail and to optimize it for clinical applications. Isolated JPCs can be sufficient for the regeneration of small bone defects. However, the biggest challenge for oral and maxillofacial surgeons is to regenerate large bone defects, as occurring after tumor resections. To avoid long in vitro culturing for the generation of high JPC numbers and the associated cell senescence, induced pluripotent stem cells (iPSCs) might serve as an alternative source of MSCs because of their unlimited self-renewal capacity and differentiation potential [7]. A variety of methods has been published to differentiate iPSCs into iPSC-derived mesenchymal stem/stromal-like cells (iMSCs), demonstrating comparable properties of MSCs and iMSCs regarding morphology, marker expression, differentiation potential, or immunomodulatory properties [8,9].

In addition, it has been reported, that iPSC-derived cells originating from fibroblasts or MSCs show signs of rejuvenation, e.g., telomere elongation, accelerated proliferation, and a rejuvenation-associated gene expression signature [10,11]. These observations raise hopes that rejuvenated iMSCs can have additional benefits over primary MSCs when used in regenerative therapies, since studies show that donor age negatively affects proliferation rates, expression of immunological factors, and cellular senescence (reviewed in [12]).

Recently, we demonstrated the generation of footprint- and xeno-free iPSCs from JPCs and their successful differentiation into iMSCs by cultivation of iPSCs with human platelet lysate (hPL)-supplemented medium [13]. We further showed that the generated iMSCs are osteogenic progenitor cells with a strong mineralization potential. We anticipate that this is due to a certain genetic memory of JPC-iMSC-derived iMSCs. 

In the present study, we compared the properties of the originating isogenic JPCs, which are themselves MSCs with a high differentiation potential [14,15,16], and the generated iMSCs, in terms of their differentiation potential and possible rejuvenation.

## 2. Results

### 2.1. Tri-Lineage Differentiation of iMSCs and JPCs

iPSCs were generated from JPCs of three different donors and differentiated into iMSCs. To demonstrate their differentiation potential, iMSCs were differentiated into the adipogenic, chondrogenic, and osteogenic lineage. Lineage-specific staining and gene expression analysis was performed and compared to the originating JPCs.

#### 2.1.1. Adipogenic Differentiation

**Figure 1:**
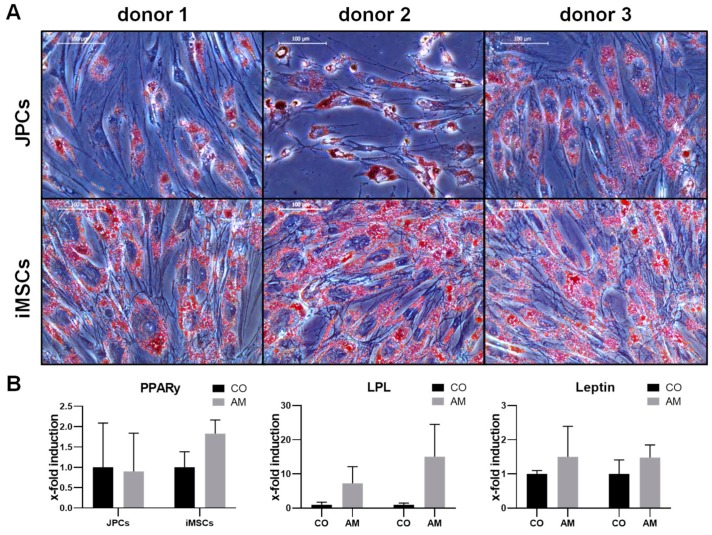
Adipogenic differentiation of jaw periosteal cells (JPCs) and iPSC-derived mesenchymal stem/stromal-like cells (iMSCs). (**A**) Microscopic images (20× magnification, scale bar = 100 µm) of oil red stained JPCs (upper panel) and iMSCs (lower panel) after 15 days (donor 3 JPCs only 10 days) of adipogenic differentiation. (**B**) Expression levels of adipogenic marker genes (*PPARγ, LPL, and leptin*) of JPCs and iMSCs cultivated for 10 days in adipogenic medium (AM) were normalized to levels of the housekeeping gene *GAPDH* and presented as x-fold induction relative to those of respective cells cultivated in control medium (CO). Differences in gene expression were compared using two-way ANOVA (*n* = 3).

JPCs and iMSCs were able to differentiate into the adipogenic lineage, as demonstrated by staining of fat vacuoles using Oil Red after 10–15 days of incubation in adipogenic medium (Figure 1A). Gene expression analysis was performed after 10 days of adipogenic differentiation, and shows an induction of adipogenic marker genes *PPARγ, LPL and leptin* in iMSCs and JPCs, yet not statistically significant (Figure 1B). A stronger oil red staining and induction of adipogenic marker genes in iMSCs was observed, however without significant differences compared to JPCs. 

#### 2.1.2. Chondrogenic Differentiation

**Figure 2:**
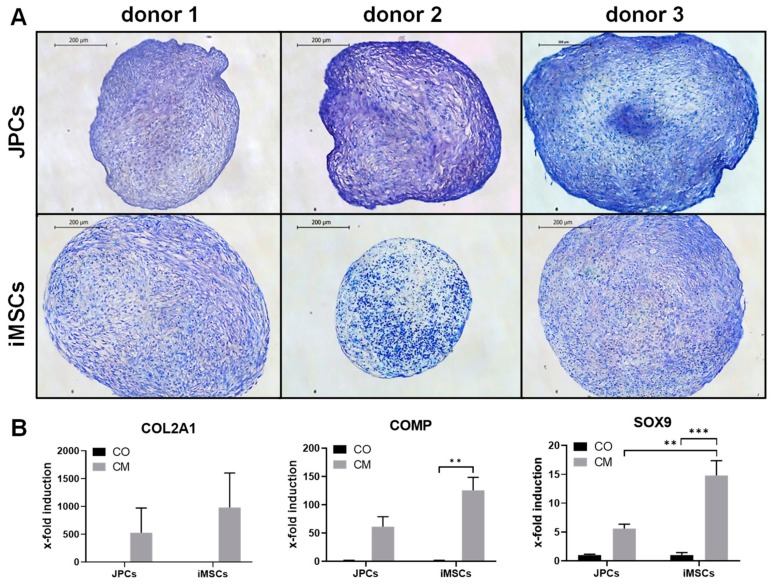
Chondrogenic differentiation of iMSCs and JPCs. (**A**) Toluidine blue staining of JPCs (upper panel) and iMSCs (lower panel) treated with chondrogenic medium (CM) for 25 days (4× magnification, scale bar = 500 µm). (**B**) Gene expression analysis (*COL2A1, SOX9, COMP*) of JPCs and iMSCs after 20 days of incubation in control (CO) and chondrogenic medium (CM). Gene expression data of JPC and iMSC samples were normalized to the corresponding expression of the housekeeping gene *GAPDH*. Gene expression mean values ± SD of CO and CM samples were displayed as x-fold induction values relative to the control samples (CO). Statistical significance was calculated using two-way ANOVA (*n* = 3 donors, ** *p* < 0.01, *** *p* < 0.001).

Chondrogenic differentiation of JPCs and iMSCs was detected by violet staining of glycosaminoglycans (GAGs) with toluidine blue after 25 days of chondrogenic differentiation. While all JPCs clearly displayed chondrogenic differentiation, iMSCs from donor 2 showed only weak differentiation (Figure 2A). iMSCs and JPCs incubated for 20 days in chondrogenic medium displayed considerable induction of chondrogenic marker genes *COL2A1*, *COMP*, and *SOX9* (Figure 2B). *COMP* (*p* < 0.01) as well as *SOX9* (*p* < 0.001) expression was significantly induced in iMSCs compared to the control samples, and a significantly higher *SOX9* induction (*p* < 0.01) in iMSCs compared to JPCs treated with chondrogenic medium was detected.

#### 2.1.3. Osteogenic Differentiation 

**Figure 3:**
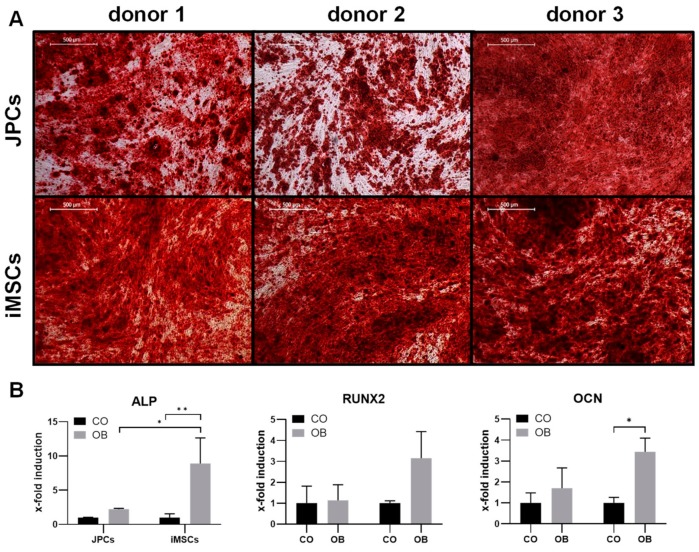
Osteogenic differentiation of iMSCs and JPCs. (**A**) Alizarin red staining of JPCs (upper panel) and iMSCs (lower panel) treated with osteogenic medium (donor 2 and 3 JPCs and iMSCs for 15 days, donor 1 JPCs 25 days, donor 1 iMSCs 20 days) (4× magnification, scale bar = 500 µm). (**B**) Gene expression analysis (*ALP*, *RUNX2*, *OCN*) of JPCs and iMSCs after 15 days of incubation in control (CO) and osteogenic medium (OB). Gene expression data of JPC and iMSC samples were normalized to the corresponding expression of the housekeeping gene *GAPDH*. Gene expression mean values ± SD of CO and OB samples were displayed as x-fold induction values relative to the CO samples. Statistical significance was calculated using two-way ANOVA (*n* = 3 donors, * *p* < 0.05, ** *p* < 0.01).

Osteogenic differentiation of iMSCs and JPCs was stimulated for 15–25 days (donor 1 JPCs: 25 days, donor 1 iMSCs: 20 days, donor 2 and 3 JPCs and iPSCs: 15 days) by incubation in osteogenic medium. Subsequently, cell monolayers were stained with alizarin red to visualize cell mineralization (Figure 3A). iMSCs and JPCs from all three donors displayed strong mineralization. Gene expression analysis shows an induction of osteogenic marker genes *ALP*, *RUNX2*, and *OCN* in iMSCs and JPCs (Figure 3B). *ALP* (*p* < 0.01) as well as *OCN* (*p* < 0.05) expression was significantly induced in iMSCs compared to the control samples. Further, *ALP* induction (*p* < 0.05) was significantly higher in iMSCs compared to JPCs, correlating with the stronger mineralization of iMSCs observed in the alizarin-stained samples.

### 2.2. Rejuvenation and Senescence

#### 2.2.1. Telomere Length Assay 

**Figure 4:**
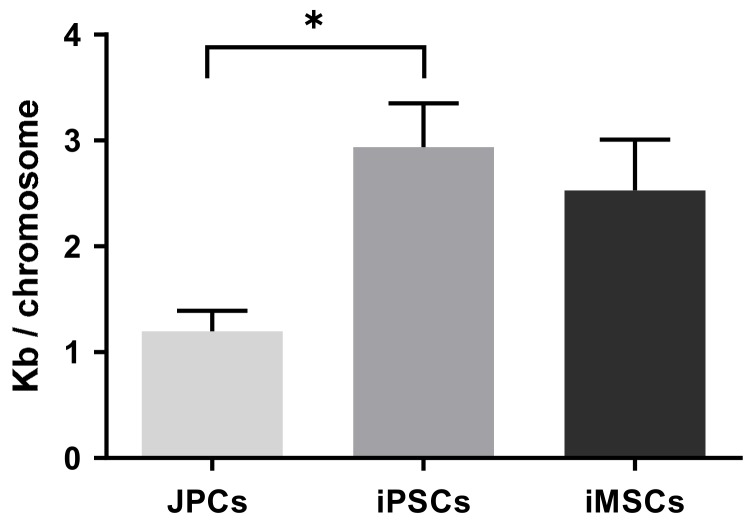
Telomere length quantification by qRT-PCR analysis of JPCs, JPC-derived induced pluripotent stem cells (iPSCs) and iMSCs (*n* = 3 donors, mean ± SEM, one-way ANOVA, * *p* < 0.05).

The determined absolute telomere length in JPC-derived iPSCs with 2.9 ± 0.4 kb/chromosome was significantly higher compared to the precursor JPCs (1.2 ± 0.2 kb/chromosome, Figure 4)). iMSCs showed a two-fold increase in telomere length (2.5 ± 0.5 kb/chromosome, Figure 4) compared to the initial JPCs, however statistically not significant. 

#### 2.2.2. Proliferation, Mitochondrial Activity, and SA-β-Galactosidase Activity

**Figure 5:**
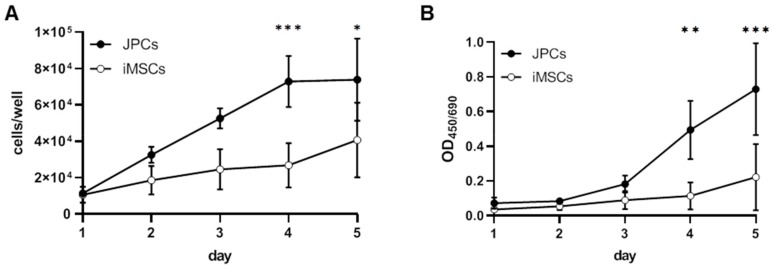
Growth kinetics and mitochondrial activity of JPCs and iMSCs. Quantification of (**A**) cell growth by cell counting and (**B**) mitochondrial activity by colorimetric measurement of formazan derivates produced by reduction of tetrazolium salts in the mitochondria of JPCs and iMSCs (EZ4U). Cell numbers and mitochondrial activities of iPSCs and JPCs were compared by two-way ANOVA (*n* = 3, * *p* < 0.05, ** *p* < 0.01, *** *p* < 0.001).

Increased telomere lengths in iPSCs and iMSCs compared to JPCs indicated a rejuvenated phenotype of obtained iMSCs. However, cell proliferation (d4: *p* < 0.001, d5: *p* < 0.05) and mitochondrial activity (d4: *p* < 0.01, d5: *p* < 0.001) were significantly lower in iMSCs compared to JPCs (Figure 5A,B). 

**Figure 6:**
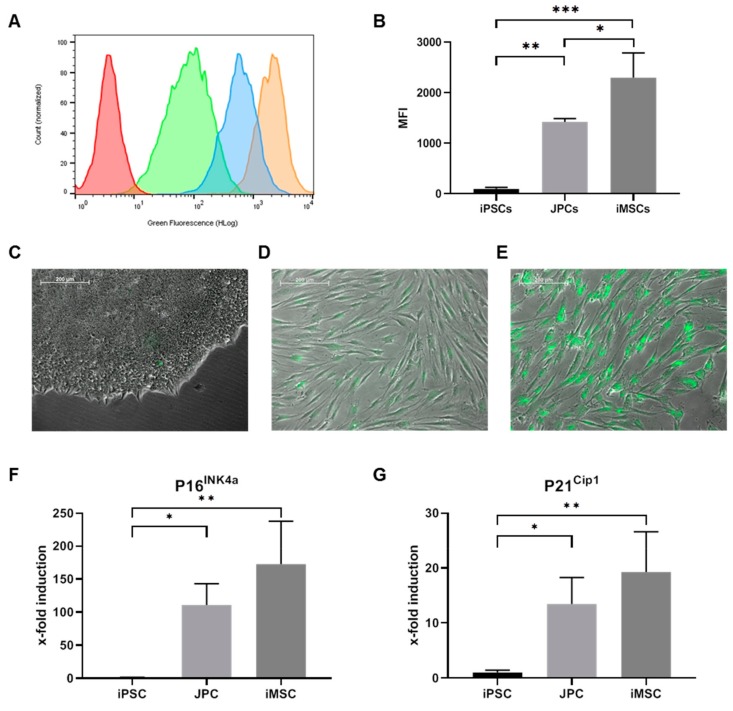
Cellular senescence of JPCs, iPSCs, and iMSCs. Determination of SA-β-galactosidase activity using a fluorescent SA-β-gal assay kit. (**A**) Representative histogram of SA-β-gal activity detected in iPSCs (green), JPCs (blue), iMSCs (orange) and an unstained control (red) by flow cytometry and (**B**) median fluorescence index (MFI) values displayed as mean values ± SD, compared by one-way ANOVA. Fluorescent SA-β-gal activity detected by fluorescence microscopy in (**C**) iPSCs, (**D**) JPCs and (**E**) iMSCs (10× magnification, scale bar = 200 µm). Expression of senescence marker genes (**F**) *P16^INK4a^* and (**G**) *P21^Cip1^* is displayed as induction values relative to the expression of iPSCs (control) and compared by one-way ANOVA (*n* = 3, * *p* < 0.05, ** *p* < 0.01, *** *p* < 0.001).

In addition, SA-β-gal activity was significantly higher in iMSCs as demonstrated in Figure 6A–E. MFI values of fluorescently detected SA-β-gal activity were significantly higher in iMSCs compared to JPCs (*p* < 0.05) and iPSCs (*p* < 0.001) (Figure 6B). Further, the expression of senescence marker genes *P16^INK4a^* and *P21^Cip1^* was higher in iMSCs compared to JPCs, however statistically not significant. Significant differences in *P16^INK4a^* and *P21^Cip1^* expression was detected in iPSCs compared to JPCs (*p* < 0.05) and iMSCs (*p* < 0.001).

## 3. Discussion

The use of iPSCs to produce clinically relevant cells for regenerative medicine is a promising perspective. Still, there are many hurdles to overcome until such cells can enter the clinical practice. To approach this aim, we established a protocol for the generation of footprint-free iPSCs and iMSCs under xeno-free conditions [13]. In the present study, we compared the resulting iMSCs with the originating isogenic JPCs concerning their three-lineage differentiation potential and investigated a potential rejuvenation of iMSCs, which has previously been observed in iPSC-derived cells [10,17]. 

Our results showed a high multipotent differentiation potential of iMSCs, demonstrating the clinical relevance of these cells for applications in regenerative medicine. Qualitative evaluation of the tri-lineage differentiation of iMSCs indicated a higher stem cell potential of iMSCs compared to JPCs, which was supported by significantly higher induction of the chondrogenic marker gene *SOX9* and the osteogenic marker gene *ALP* in iMSCs compared to the originating JPCs.

In addition, an increased telomere length was found in iPSCs and iMSCs. Telomere elongation was shown to be a consequence of reprogramming caused by telomerase activation in iPSCs [18]. The two-fold higher telomere length detected in iMSCs compared to JPCs indicated a higher proliferative capacity of these cells, as telomere elongation had been demonstrated to enable cells to overcome replicative senescence [19].

To examine possible rejuvenation in the generated iMSCs, growth rates, mitochondrial activities, and SA-β-gal activities were investigated. Surprisingly, iMSCs showed lower proliferation rates and mitochondrial activities compared to JPCs. In addition, iMSCs expressed higher levels of the senescence marker genes *P16^INK4a^* and *P21^Cip1^*, as well as higher SA-β-gal activities, clearly displaying an early senescence phenotype. However, as telomeres were elongated in iMSCs compared to JPCs, replicative senescence can be excluded as a possible reason for this phenotype. 

Similar to our findings, Feng et al. observed early senescence in iPSC-derived hemangioblast cells and assumed an influence of retroviral vector insertion and reactivation of reprogramming genes during iPSC differentiation [20]. This theory was refuted by a study from Gokoh et al. demonstrating that the avoidance of stressful conditions, and a more efficient protocol using additional cytokines for hemangioblast differentiation, could overcome these issues [21]. We also hypothesize that cellular stress caused by the differentiation process could induce senescence in iMSCs. This assumption is supported by the observation of massive cell death after single-cell plating of iPSCs, and later transfer to uncoated cell culture dishes, during iMSC differentiation. A mechanism possibly underlying this senescence phenotype could be the stress-induced production of reactive oxygen species and subsequent activation of the *P16^INK4a^* pathway, caused by the disruption of iPSC colonies, similar to tissue damage-induced senescence [22,23]. Further, we discovered that higher iMSC yields could be achieved by using iPSCs that had not been passaged for 10 or more days, which was possibly priming iPSC differentiation by high cell densities. Therefore, we consider iMSC differentiation by this protocol a highly selective process that favors cells that are already differentiated or in which differentiation has been primed prior to separation. 

Devito et al. also observed senescent cells during iMSC differentiation when using a similar method with single-cell plating and incubation of separated iPSCs in MSC medium containing hPL [24]. Interestingly, they did not observe senescent cells when using a protocol in which iPSCs were first incubated with the TGF-β signaling inhibitor SB-431542 to induce differentiation for 20 days, before separation and selection of iMSCs in MSC medium [24].

Thus, we assume that iMSC generation by single-cell plating of iPSCs and subsequent incubation in serum containing MSC media, as performed in this study and as suggested by several recent publications [25,26], is not sufficiently effective and needs to be further optimized to allow the generation of high-quality iMSCs for clinical applications. We are convinced that this can be achieved by improving efficiency and reducing cellular stress during iMSC differentiation, for example by introducing differentiation stimulating factors like SB-431542, and by avoiding separation of undifferentiated iPSCs. 

Furthermore, iMSCs are promising since they show less sensitivity to INF-γ stimulation as described in the literature [27]. Our generated iMSCs reveal also a clear insensitivity to IFN-γ-mediated HLA-DR expression compared to the JPCs, as shown in the Supplementary Figure S1. In further studies, immunomodulatory functions of generated iMSCs would be of interest in order to assess their clinical applicability. Among others, Rap-1, as a novel modulator of NF-κB activity and as a key regulator of paracrine activities of MSCs [28], should be investigated. 

## 4. Materials and Methods 

### 4.1. Cell Culture

JPCs derived from three donors were included in this study in accordance with the local ethical committee (approval number 074/2016BO2) and after obtaining written informed consent. Jaw periosteal tissue was extracted during routine surgery and JPCs were isolated and expanded as previously reported [13]. JPCs and iMSCs were grown in hPL5-medium (DMEM/F12 (Gibco) + 5 % human platelet lysate (hPL, ZKT Tübingen GmbH), 100 U/mL penicillin-streptomycin (Pen-Strep, Lonza, Basel, Switzerland), 2.5 µg/mL amphotericin B (Biochrom, Berlin, Germany)). 

iPSCs were maintained in Essential 8 medium (E8, Thermo Fisher Scientific Inc., Waltham, MA, USA) with daily medium changes and passaged every 4–6 days using 10 µM Y27632 ROCK inhibitor (Selleck Chemicals LLC, Houston, TX, USA).

### 4.2. Generation of Integration-Free iPSCs from JPCs Using srRNA

JPCs were reprogrammed to iPSCs as previously published [13]. Briefly, JPCs were incubated in hPL5 medium containing 0.2 µg/mL recombinant B18R protein (eBioscience, San Diego, CA, USA) prior to transfection with a self-replicating VEE-OKSM-GFP RNA. From day 1–5 transfected cells were incubated with hPL5 + 25% B18R conditioned medium (BcM) + 1 µg/mL puromycin (Invivogen, Toulouse, France) to select transfected cells. Total of 250 µM histone deacetylase inhibitor sodium butyrate (NaB, Selleck Chemicals LLC, Houston, TX, USA) was added to the medium to enhance reprogramming efficiency. At day 7, the medium was changed to E8 medium (Essential 8, Thermo Fisher Scientific Inc., Waltham, USA) + 25% BcM. Single iPSC colonies were picked and transferred into VTN-coated wells containing E8 medium + 10 µM Y27632 ROCK inhibitor (Selleck Chemicals LLC, Houston, TX, USA) and maintained in E8 medium with daily medium changes. Passaging was performed every 4–6 days.

### 4.3. Differentiation of iPSCs to iMSCs

Very low concentrations of iPSCs (≤ 10% confluency) were seeded into VTN-coated 12-well plates and cultivated in E8 medium without passaging for 10 days to stimulate spontaneous differentiation. After this period, the cells were detached using Accutase and transferred into VTN-coated 6-well plates containing E8 medium and 10 µM ROCK inhibitor Y27632 (passage 0). The next day, the medium was changed to hPL5 + 150 µM L-ascorbic acid 2-phosphate (Sigma-Aldrich, St. Louis, MO, USA) and replaced every other day. After reaching 80% confluency, cells were passaged (split ratio 1:3) using Accutase. ROCK inhibitor was added to the medium for 24 h after passaging. For following cell passaging TrypLE Express was used and no further ROCK inhibitor was added. Cells were passaged until the morphology of the cells had changed to a spindle-shaped MSC-like appearance (3–5 passages). Cells exhibiting MSC morphology were expanded in hPL5 prior to further experiments. 

### 4.4. Tri-Lineage Differentiation of iMSCs

#### 4.4.1. Osteogenic Differentiation 

To stimulate osteogenic differentiation, iMSCs were cultivated in osteogenic medium (DMEM/F12 + 10% hPL, Pen-Strep, amphotericin B, 0.1 mM l-ascorbic acid 2-phosphate (Sigma-Aldrich, St. Louis, MO, USA), β-glycerophosphate (AppliChem, Darmstadt, Germany), 4 µM dexamethasone (Sigma-Aldrich, St. Louis, MO, USA)) with medium changes every other day. After 15–25 days, cells were fixed with 4% formalin and monolayers were stained with 1 mL of Alizarin red solution (40 mM, pH 4.2) for 20 min. Unbound dye was washed off with dest. water and images were taken using an inverted microscope (Leica, Wetzlar, Germany).

#### 4.4.2. Adipogenic Differentiation 

For adipogenic differentiation, 5×10^4^ cells/well were seeded into 12-well plates and incubated with adipogenic medium (AM) containing (DMEM/F12 + 10% hPL, Pen-Strep, amphotericin B, 0.2 mM indomathacin (Sigma-Aldrich, St. Louis, MO, USA), 0.5 mM 3-isobutylxanthin (Sigma-Aldrich, St. Louis, MO, USA), 1 µM dexamethasone (Sigma-Aldrich, St. Louis, MO, USA)) 10 µg/mL insulin (Sigma-Aldrich, St. Louis, MO, USA).

RNA isolation for gene expression analysis was performed at day 10. After 10–15 days, cells were fixed with 4% formalin and stained with 0.5 mL of a 3 µg/mL oil red O solution (Sigma-Aldrich, St. Louis, MO, USA) containing 60% isopropanol for 15 min. Unbound dye was washed off with dest. water and images were taken using an inverted microscope (Leica, Wetzlar, Germany). 

#### 4.4.3. Chondrogenic Differentiation 

For chondrogenic differentiation, cells were harvested and resuspended in control medium (CO, hPL5) and chondrogenic medium (CM) containing DMEM high glucose (4,5 g/L) + pyruvate (1 mM) (Thermo Fisher Scientific Inc., Waltham, USA), 1 % ITS+ Premix (Corning Inc., New York, USA), 100 U/mL Pen-Strep (Lonza, Basel, Switzerland), 2.5 µg/mL amphotericin B (Biochrom, Berlin, Germany), 0.1 µM L-ascorbic acid 2-phosphate (Sigma-Aldrich, St. Louis, MO, USA), 0.1 µM dexamethasone (Sigma-Aldrich, St. Louis, MO, USA), and 10 ng/mL TGF-β1 (PeproTech, Hamburg, Germany) at a concentration of 1.25 × 10^6^ cells/mL. Total of 0.2 mL/well of the cell suspension (0.25×10^6^ cells) was added into a low adherence V-bottom polypropylene 96-well plate and centrifuged for 5 min at 350× *g* to form pellets. The medium was changed every other day. On day 20, pellets were harvested for gene expression analysis. After 25 days, pellets were fixed with 4% formalin, embedded in paraffin, and cut with a microtome. Slices were stained using a 0.04 % toluidine blue staining solution.

### 4.5. Mitochondrial Activity Assay 

To compare the mitochondrial activities of iMSCs and JPCs, the EZ4U kit (Biomedica, Vienna, Austria) was used. Proliferation measurements were performed on five consecutive days in intervals of 24 h, following the manufacturer’s instructions. Briefly, 1.33 × 10^3^ cells/well were seeded into 96-well plates containing 0.2 mL hPL5 on day 0. After the respective incubation times (1–5 days), the medium was replaced by 200 µL medium + 20 µL of pNPP-substrate, and the mixture was incubated for 4 h at 37 °C. Subsequently, absorption was measured at a wavelength of 450/620 nm using an ELx800 ELISA Reader (Bio-Tek, Winooski, VT, USA). KC4 software was used for data evaluation.

### 4.6. Growth Kinetics

2.5 × 10^4^ cells were seeded into 12-well plates containing hPL5 in triplicates and harvested for cell counting in an interval of 24 h on five consecutive days. After trypsinization, cells of three wells were pooled, centrifuged, and resuspended in PBS. To identify the dead cells, cell suspensions were incubated with 10 µg/mL propidium iodide (Sigma-Aldrich, St. Louis, MO, USA) for 1 min before cell counting. Subsequently, cell concentrations were measured using the Guava EasyCyte 6HT-2L flow cytometry instrument (Merck Millipore, Billerica, MA, USA).

### 4.7. Senescence Assay

To assess the cellular senescence, beta-galactosidase activity was detected using the Senescence Assay Kit (Abcam, Cambridge, UK) following the manufacturer’s instructions. Briefly, 2.5 × 10^4^ cells were seeded in triplicates into 12-well plates containing hPL5. After 48 h, fresh hPL5 medium containing 3 μL/mL of senescence dye was added to the cells and incubated for 1–2 h. After incubation, fluorescence microscopic images were taken using an Axio Observer.Z1 microscope and AxioVision 4.8.2 software (Carl Zeiss, Oberkochen, Germany). Subsequently, cells were detached, pooled, and resuspended in wash buffer (included in the kit) and flow cytometry measurements were performed using the Guava EasyCyte 6HT-2L flow cytometry instrument (Merck Millipore, Billerica, MA, USA).

### 4.8. Telomere Length Measurement

The telomere length of JPCs, JPC-derived iPSCs, and iMSCs was analyzed using absolute human length quantification qRT-PCR Assay Kit (ScienCell, Carlsbad, CA, USA) according to the manufacturer’s instructions. Therefore, genomic DNA was isolated using QIAamp DNA Mini Kit (Qiagen, Hilden, Germany). qRT-PCR reactions were performed in CFX Connect Real-Time PCR Detection System (Bio-Rad) using IQ™ SYBR^®^Green Supermix (Bio-Rad) and 10 ng gDNA. A genomic DNA sample with known telomere length served as reference for target sample quantification and a single copy reference was used for data normalization.

### 4.9. Gene Expression Analysis of JPCs and iMSCs

RNA isolation from JPCs and iMSCs was performed using the NucleoSpin RNA kit (Macherey-Nagel, Düren, Germany) following the manufacturer’s instructions. RNA concentration was measured using a Qubit 3.0 fluorometer and the corresponding RNA BR Assay Kit (Thermo Fisher Scientific Inc., Waltham, MA, USA). Total of 0.5 μg of RNA was used for first-strand cDNA synthesis using the SuperScript Vilo Kit (Thermo Fisher Scientific Inc., Waltham, MA, USA). The quantification of mRNA expression levels was performed using the real-time LightCycler System (Roche Diagnostics, Mannheim, Germany). For the PCR reactions, commercial PPARγ, LPL, Leptin, COL2A1, COL10A1, SOX9, COMP, ALP, RUNX2, OCN, COL1A1 primer kits (Search LC, Heidelberg, Germany), and DNA Master SYBR Green I (Roche, Basel, Switzerland) were used. The amplification was performed with a touchdown PCR protocol of 40 cycles (annealing temperature between 68–58 °C), following the manufacturer’s instructions. Copy numbers of each sample were calculated on the basis of a standard curve (standard included in the primer kits), and normalized to the housekeeping gene glyceraldehyde-3-phosphate dehydrogenase (*GAPDH*). X-fold induction values were calculated by the quotient of the sample and the corresponding control (iPSCs).

### 4.10. Statistical Analysis

For the evaluation of differentiation marker gene expression, cell growth and mitochondrial activity data, means ± standard deviations were calculated and compared by two-way ANOVA (*p* adjusted using Sidak’s multiple comparison test) using GraphPad Prism 8.1.0 software. Mean ± standard deviations of senescence marker gene expression was compared by one-way ANOVA (*p* adjusted using Tukey’s multiple comparison test). To analyze the telomere lengths and SA-β-Gal expression (MFI), means ± SD were calculated and compared by one-way ANOVA (*p* adjusted using Tukey’s multiple comparison test). A *p*-value ≤ 0.05 was considered significant.

## 5. Conclusions

This study showed a high potential of footprint- and xeno-free generated iMSCs to differentiate into bone, cartilage, and fat in vitro, demonstrating the clinical relevance of this cell type. However, the quality of the resulting cells was not satisfactory because of the observation of early senescence. Revised protocols should focus on raising differentiation efficiency and avoiding cellular stress to increase iMSC quality.

## Supplementary Materials

Supplementary materials can be found at https://www.mdpi.com/1422-0067/21/2/587/s1. Figure S1: HLA-I and HLA-II expression of JPCs and iMSCs

## Abbreviations

AM	adipogenic medium
ANOVA	analysis of variance
BcM	B18R-conditioned medium
CM	chondrogenic medium
CO	control
FBS	fetal bovine serum
GAGs	glycosaminoglycans
GFP	green fluorescent protein
hPL	human platelet lysate
iMSC	iPSC-derived mesenchymal stem/stromal like cell
iPSC	induced pluripotent stem cell
JPC	jaw periosteal cell
kb	kilo basepairs
MFI	median fluorescence index
mRNA	messenger RNA
MSC	mesenchymal stem/stromal cell
NaB	sodium butyrate
OB	osteoblast medium
OSKM	OKT4, SOX2, KLF4, cMYC
PBS	phosphate buffered saline
PCR	polymerase chain reaction
Puro	puromycin
qRT-PCR	quantitative real-time polymerase chain reaction
ROCK	rho-associated protein kinase
SA-β-gal	senescence-associated beta-galactosidase
SD	standard deviation
SEM	standard error of mean
srRNA	self-replicating ribonucleic acid
