# Supplementary material for: iPSC-Derived MSCs Versus Originating Jaw Periosteal Cells: Comparison of Resulting Phenotype and Stem Cell Potential
Source: Int J Mol Sci. 2020 Jan 16;21(2):587. doi: 10.3390/ijms21020587 (PMC7013802; doi:10.3390/ijms21020587)
Supplement: Supplementary file 1 [file ijms-21-00587-s001.pdf]

**Supplementary Materials:** Figure S1: HLA-I and HLA-II expression of JPCs and iMSCs.

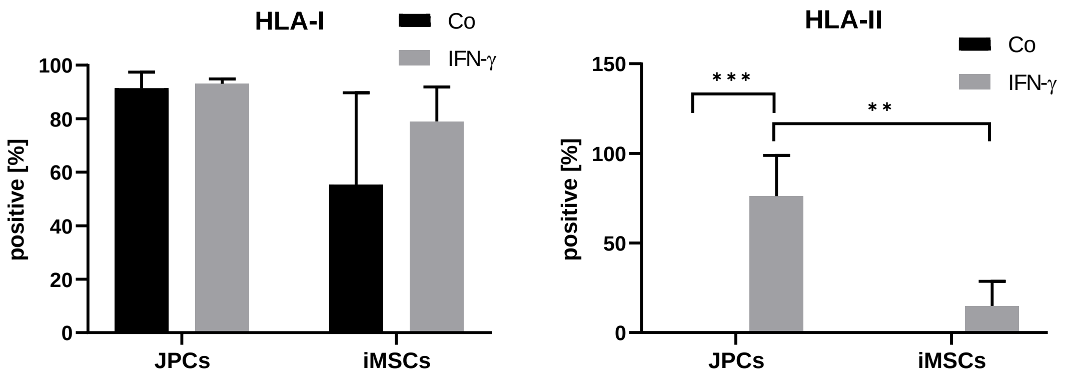


**Figure S1.** HLA-I and HLA-II expression of JPCs and iMSCs of untreated samples (Co) and after stimulation with 200 ng/ml IFN-γ (Sigma-Aldrich, St. Louis, USA) for 7 days (IFN-γ). Surface marker expression was detected by flow cytometry using APC-conjugated anti-HLA-I and anti-HLA-II antibodies (Biolegend, San Diego, USA). Positive cells were gated on the basis of the corresponding isotype control samples. Differences in surface marker expression were compared using two-way ANOVA (n = 3 patients, ** = $p < 0.01$, *** = $p < 0.001$).
